# Creativity, Boredom Proneness and Well-Being in the Pandemic

**DOI:** 10.3390/bs12030068

**Published:** 2022-03-02

**Authors:** Nicholaus P. Brosowsky, Nathaniel Barr, Jhotisha Mugon, Abigail A. Scholer, Paul Seli, James Danckert

**Affiliations:** 1 Department of Psychology, University of Manitoba, 190 Dysart Rd., Winnipeg, MB R3T 2N2, Canada; nbrosowsky@gmail.com; 2 Faculty of Humanities and Social Sciences, Sheridan College, 1430 Trafalgar Rd., Oakville, ON L6H 2L1, Canada; nathanial.barr@sheridancollege.ca; 3 Department of Psychology, University of Waterloo, 200 University Avenue West, Waterloo, ON N2L 3G1, Canada; jmugon@gmail.com (J.M.); ascholer@uwaterloo.ca (A.A.S.); 4 Department of Psychology and Neuroscience, Duke University, 417 Chapel Drive, Durham, NC 27708, USA; pseli@gmail.com

**Keywords:** creativity, boredom proneness, COVID-19 pandemic, well-being

## Abstract

Throughout the course of the pandemic, it has become clear that the strictures of social isolation and various levels of lockdown constraints have impacted people’s well-being. Here, our aim was to explore relations between trait dispositions associated with boredom proneness, self-regulation and well-being using data collected early in the pandemic. Specifically, we explored whether the tendency to engage in everyday creative pursuits (e.g., making your own greeting cards) would act as a prophylactic against poor well-being. Results showed that well-being was higher for those individuals who increased engagement with creative pursuits during the early stages of the pandemic. That is, people who engaged more in everyday creative activities also reported higher levels of self-esteem, optimism, and positive affect. In contrast, those who pursued fewer creative outlets had higher levels of depression and anxiety, were higher in boredom proneness, and reported experiencing more negative affect. As we emerge from the pandemic, these data provide a clue as to how people might plan to cope adaptively with the restrictive circumstances this extreme world event engendered. More generally, these data provide support for the notion that everyday creativity (and not necessarily creative expertise) has positive associations for well-being.

## 1. Introduction

The COVID-19 global pandemic has wrought not only severe public health challenges, but large-scale societal, organizational, economic, and personal ones. The constraints imposed on our daily lives in the service of limiting the spread of coronavirus has resulted in individuals being confined to their homes and a marked disruption of normal activities. The imposition of these extraordinary restrictions on human movement and activity presents an opportunity for psychologists to examine the influence of various trait dispositions and behaviours on the capacity to adhere to these rules and to cope with their consequences. In this way, psychologists can contribute to public health policy by better understanding the extent to which such factors interact in the dynamic reality of human experience.

Of particular concern are the challenges that lockdowns and other constraints on social interaction pose for mental health [1]. In a study of adults in the UK with data collected over multiple time points, results showed that suicidal ideation and anxiety increased over time during the pandemic [1]. It is worth noting that rates of depressive symptoms were unchanged. In another study, it was found that rates of cognitive failures (e.g., forgetfulness and mind-wandering) increased early in the pandemic [2]. Interestingly, this was mitigated somewhat by the extent to which participants reported being mindful. Clearly, mental health is a serious concern during these extraordinary times [3,4]. In addition, boredom proneness—the tendency to experience boredom more frequently and intensely—has been shown to be related to poor adherence to the strictures of social isolation [5,6]. Taken together, these results suggest that the constraints imposed by the pandemic place a burden of self-regulatory capacities which in turn is likely to have negative impacts on well-being [7,8,9].

While an individual’s pre-existing mental health challenges, socioeconomic barriers [1], and certain individual traits (e.g., boredom proneness, self-control) [5,6] represent clear obstacles to well-being in these times, less is known about factors that might *promote* well-being. One such factor may be one’s level of engagement in creative outlets or pursuits. At various stages of the pandemic, a variety of activities have emerged as salves to the constraints imposed by lockdowns, from learning a new language, to sourdough baking, to jigsaw puzzles, and beyond [10,11,12]. In addition, the presence of natural elements (e.g., sunlight, potted plants, a view of green space [13]) has been associated with mitigation of negative affective responses to lockdown measures (e.g., less anxiety, fear, and even boredom). Critically, the pandemic may well have had a strong influence on how people perceive both their own well-being and the nature of what counts as a creative outlet. In particular, the pandemic may have elevated the experience of boredom to the forefront of people’s minds, casting it as a more “normative” experience, while at the same time thrusting the need for creative outlets (or access to greenspace as in [13]) for action into stark relief. Here, we examined the hypothesis that engaging in everyday creative activities may act to promote well-being in the context of the challenges of the COVID-19 pandemic.

To clarify our terms, we consider creative activities to broadly encompass self-initiated actions or goals that allow the individual to discover or produce something novel (to them) or that allows them to express some emotion or feeling state [14]. This is intentionally broad as it encompasses things as diverse as sourdough baking (a popular activity during the early stages of the pandemic) [9] and cultivating green space [13], as well as activities more traditionally considered under the term “creative” (e.g., painting, sculpting, writing, etc.). With respect to well-being, we employed metrics of affective experience and trait dispositions known to be associated with mental health (see below). We take as the basis of our definition of well-being the APA dictionary definition of “a state of happiness and contentment, with low levels of distress, overall good physical and mental health and outlook, or good quality of life” [14]. We recognize that our label, “well-being”, and our chosen metrics, may reflect either an individual’s capacity to cope with the constraints of the pandemic or some general form of life satisfaction (or both). Where possible, we indicate which of those facets we believe our results pertain to.

The capacity for creativity has long been associated with both mental illness *and* well-being [15,16,17,18]. On the one hand there are suggestions that elevated levels of arousal, characteristic of mania, are commonly associated with creativity, and that novel connections necessary for creative expression are more common to some mental illnesses [19]; see [16] for a review. Historically, much has been made of famous artists and their struggles with mental health [20,21]. In addition, research has long sought to establish a link between creativity and the psychological condition of schizophrenia [22,23]. In a recent meta-analysis of this work, results suggested that an inverted-U function best described the relation, with mild to moderate levels of severity in schizophrenia being associated with an increase in creativity, while more severe forms of the disease were associated with decreased creativity. On the other hand, research has focused on the benefits of creative outlets both in terms of the therapeutic value of creative engagement for those suffering from mental illnesses and as a more general benefit to healthy individuals [24]. With respect to the association between mental illness and creativity, it is perhaps worth pointing out that much of this theorizing centers on individuals who have attained unusually high proficiency in a given domain [16]. Others have suggested that creativity lies on a continuum and that “everyday” creativity may play an important role in well-being e.g., [25].

In the context of the pandemic, creativity has been associated with a number of other factors that may make it more or less likely to promote well-being. Creativity is associated with a tendency towards nonconformity, divergent thinking, and the capacity to break with social norms [16]. Such tendencies are in direct contrast with the constraints imposed during the pandemic of social isolation and, in the extreme, lockdowns. In contrast, creativity is also associated with openness to experience, playfulness, cognitive flexibility, and a willingness to try new things [26]—all of which are factors that might be expected to positively influence one’s capacity to cope with the constraints of the pandemic. Indeed, recent research examining the relation between engagement in creative activities, positive and negative affect, and flourishing, found that following days featuring more creative activity than usual (in typical circumstances), people reported higher positive affect and flourishing [27]. Interestingly, one recent study suggested that the therapeutic benefits of creative outlets stem from the fostering of playfulness and inspiration, independent of any influence of the social aspects involved in the activity [26]. Thus, the restrictions inherent to the pandemic provide a unique opportunity to examine this account of the therapeutic import of creativity.

It is worth noting that the kinds of activities we are referring to here are not exclusively or even necessarily those we normally think of when using the term “creative”. That is, it is not necessary that the activity be one traditionally conceived of as part of the arts—sculpting, painting, or creative writing, for example. Making a friend a birthday card, for instance, or crafting an object from various materials simply for the sake of expressing oneself, count as examples of being creative. Furthermore, expertise may be less crucial than simply exercising creative processes.

Some research has examined the influence of lockdown measures on creativity, finding that everyday creativity increased, particularly amongst those with a lower baseline of creative activity [28]. Among students, one study showed a slight increase in creative activity during the pandemic [29]. This study also showed that higher levels of creativity were associated with increased levels of reported positive affect. Furthermore, it is plausible that the constraints of the pandemic have had an influence not only on the number of creative activities engaged in, but also on their form. What is lacking here is a broader understanding of the outcomes associated with creativity as a function of the constraints inherent to the pandemic.

We asked people to respond to a range of questionnaires during the early stages of the pandemic (late April, early May 2020) [5]. Within the larger dataset reported in [5], participants were asked to report on the extent to which they engaged in creative pursuits, and they filled out a range of questionnaires that broadly examined aspects related to well-being, including self-esteem, boredom proneness, general feelings of optimism, and ratings of positive and negative affect. We also asked people about the nature of the lockdown measures evident in their particular region. We approached this by asking whether essential or nonessential businesses were closed, travel had been restricted, and whether they were asked to stay isolated at home or were allowed outside. Answers were given on a four-point scale: 0 = not at all; 1 = optional; 2 = strongly enforced; 4 = mandatory. In general, the severity of lockdown measures was associated with negative outcomes on all metrics of well-being. However, when added to our regressions they did not substantially alter the results. Our aim here was to explore the extent to which engaging in creative outlets would act to bolster well-being during these extraordinary times. More specifically, we expected that engaging in more creative outlets would be associated with better well-being.

## 2. Materials and Methods

Our sample has been described elsewhere [5] and is available publicly (https://osf.io/dmk9s/, accessed on 9 June 2021). In short, participants were recruited via Amazon’s Mechanical Turk (MTurk) and to be eligible had to have a 95% HIT acceptance rate (a metric of the MTurker’s reliability) and have completed >500 HITs (human intelligence tasks). The sample consisted of 993 individuals. Data from 69 participants were removed as they were identified as nonserious responders (7% of total cases). To determine whether a participant was a valid responder or not we first conducted reliability analyses with participants set as items. Participants with item-total correlations less than 0.20 were removed as nonserious responders (overall participant reliability; Cronbach’s α = 0.994). Next, we used a number of attention checks to ensure the integrity of our data. These included a short math question (“What is 20% of 400?”), asking participants to type the sentence “bot not am I” in the reverse order, and having participants respond to a simple question (e.g., “Should government-issued ID be required to vote in elections?”). This final question was inspected for nonsensical answers as a potential flag for nonserious responses. For example, responses of the ilk of “excellent” to this open-ended question were considered a red flag of a possible bot responder [5]. The final sample consisted of 924 participants (386 female, 530 male, 8 responding as “other” gender; age range = 18–77 years; mean age 37.70 years, *SD* = 11.25; see [5] for breakdown of ethnicities). To estimate our sensitivity to detect various effect sizes, we used the ‘WebPwer’ R package [30], which calculates the power to detect changes in R^2^ across two linear regression models in terms of Cohen’s f^2^. We estimated our ability to detect changes in R^2^ between a model containing four predictors and the intercept-only model. With 924 participants, we were well-powered (~94%) to detect an f^2^ of 0.02. Cohen suggests that f^2^ values of 0.02, 0.15, and 0.35 represent small, medium, and large effect sizes, respectively [31]. Thus, with our sample size we were well-powered to detect relatively small effect sizes.

The sample came primarily from the United States (*n* = 913), with the data collected between 28 April and 2 May of 2020.

### 2.1. Questionnaires

Participants completed a large number of questionnaires of which a subset of responses are reported here. The full set of questionnaires and dataset can be accessed at https://osf.io/dmk9s/, accessed on 9 June 2021).

### 2.2. Available Activities Pre-Pandemic

Whether the pursuit of creative activities would act as a positive or negative influence on well-being may well be influenced by the sheer number of potential outlets people had available prior to the pandemic. That is, those with a wide array of potential outlets for engagement—creative or otherwise—may have their mental health more adversely affected by the removal of those outlets during the pandemic. Conversely, if there were few outlets for creative engagement prior to the pandemic, there may be little effect of creativity on well-being post pandemic lockdowns.

We addressed this possibility in two ways. First, we examined the relation between the number of activities available across a range of domains, reasoning that the sheer availability of activities might influence well-being. We asked people to describe the available activities across the following domains: (1) exercise activities including gyms and sports, (2) work opportunities, (3) places to “hang out” (e.g., cafes, parks), (4) social activities (e.g., clubs), and (5) interesting or fun businesses (e.g., bars, restaurants, movie theatres), as well as an overall rating of available activities in the participant’s area. This metric clearly does not assess whether or not people actually availed themselves of those activities on a regular basis, but merely gave us some insight into the range of possible activities they could have availed themselves of had they chosen to. These measures were then rated on a visual analogue slider scale with anchors of few-to-none available (0), moderate availability (25), good availability (50), very good availability (75), and excellent availability (100).

Second, we created a measure of the extent to which people engaged in creative outlets *after* the onset of the pandemic *relative to their engagement in those same activities prior to the pandemic* by creating a difference score (see below). Here, we reasoned that a positive difference score (i.e., indicative of *increased* engagement in creative outlets) would be associated with higher ratings of well-being and vice versa.

### 2.3. Creative Behaviours Inventory (CBI)

Participants completed the Creative Behaviours Inventory [32], which asks people to rate a range of statements of activities and accomplishments commonly considered to be creative on a four-point Likert scale (“never did this” at one end of the scale to “more than 5 times” at the other end) when reflecting on the frequency of their behavior across adolescence and adulthood. Recent research critically examining the psychometric properties of the 28-item inventory found it to have excellent reliability (Cronbach’s alpha of 0.91) and suitable dimensionality and item discrimination, leading to the conclusion that the CBI is a useful tool for apprehending the everyday creative behavior of individuals [33].

In addition to the CBI, we asked participants to indicate the extent to which they had engaged in creative activities *over the past week* as a more recent measure of creative engagement during the pandemic. Participants responded on a five-point scale with anchors of not at all (1), rarely (2), sometimes (3), often (4), and constantly (5).

To examine *change* in creative behaviours as a function of the pandemic, we created a difference score by first normalizing the creative behaviours a participant reported engaging in over the previous week (i.e., during the pandemic measured with the single item outlined above). A participant’s CBI score was then taken to reflect *typical* engagement in creative endeavours *prior* to the pandemic. Subtracting the CBI from the normalized score reflective of the past week’s creative behaviours provides an indication of change in engagement in creative pursuits with positive scores indicating an increase in creative behaviours, and vice versa.

### 2.4. Measures of Well-Being

We used a range of scales as measures of well-being. First, to measure general levels of affect we had participants complete the positive and negative affect schedule (PANAS) [34]. The PANAS presents people with a list of words (e.g., nervous, attentive, upset, etc.) which are rated in terms of the extent to which the participant feels those words reflect how they have generally felt over the previous week. To more directly examine depression, anxiety and stress, participants completed the Depression, Anxiety, and Stress Scale (DASS) [35,36]. The DASS is an accepted measure of symptoms of depression, anxiety, and stress with items such as “I find it hard to wind down”, and “I felt down-hearted and blue” rated on a four-point scale in terms of how they related to the participant over the past week (1 = does not apply to me; 4 = applies to me very much, or most of the time). Although individual trait measures are generally assumed to be stable over time, we included several trait measures that would be considered relevant to well-being. The first of these was the Rosenberg Self-esteem Scale (RSES) [37], on which higher levels of self-esteem are assumed to be related to better well-being, e.g., [38]. The RSES includes items such as “I certainly feel useless at times” rated on a nine-point scale (1 = very strongly disagree; 9 = very strongly agree) based on how participants generally feel. As a measure of general optimism, we used the Life Orientation Test-revised (LOT-r) [39]. The LOT-r includes items such as “In uncertain times, I usually expect the best” rated on a five-point scale (1 = I agree a lot; 5 = I disagree a lot). Again, higher levels of optimism were assumed to be associated with higher well-being (e.g., [40]). Given prior work showing that boredom proneness was associated with rule-breaking in the pandemic [5,41], and the oft cited association between state boredom and creativity [42,43] (although, see [44] for a counterargument to this association), we also included responses on the shortened Boredom Proneness Scale (SBPS) [45]. That is, a handful of studies have suggested that state boredom leads to increased creativity [42,43]. Although we do not have a measure of state boredom, work has shown that boredom proneness is associated with increased frequency and intensity of experiencing the state [46]. As such, it may be the case that the boredom-prone also engage more in creative activities in response to frequent bouts of state boredom. It should be noted that the opposite direction of relation is also plausible given prior work showing that boredom prone individuals fail to launch into action [47]. This possibility is strengthened by research showing that boredom proneness is commonly only associated with poor mental health outcomes (see [44,48] for reviews), suggesting that in this instance, highly boredom-prone individuals would exhibit poor well-being and may therefore be less likely to engage in creative endeavours. The SBPS includes items such as “I find it hard to entertain myself” rated on a seven-point scale (1 = strongly disagree; 7 = strongly agree).

As we state above, prior research on creativity might suggest that those who foster creative outlets also tend to be non-conformist. As such, we included our measure of rule-breaking during the pandemic. This was a composite based on factor loadings derived from questions probing the extent to which individuals had adhered (or not) to the rules of social distancing (e.g., having social gatherings with people outside of your immediate household; see [5] for a full description of this facet of the data). The rule-breaking factor was examined via nine questions that asked such things as the extent to which people had attended social gatherings both inside and outside their homes, and the extent to which they had practiced (or not) measures of social distancing. A factor analysis revealed a single factor made up of seven of the nine questions (i.e., questions relating to hand-washing behaviour and the number of days spent in isolation did not load onto the factor and so were not used in creating factor loadings).

Clearly, we could have chosen any number of metrics related to an individual’s well-being. Some were chosen as well-validated measures of symptoms of mental illness (i.e., the DASS), while others provide good insights into current mental states (i.e., the PANAS). Our trait measures were driven in part by the research focus in the Danckert lab on boredom proneness. That is, prior research has shown relations between boredom and self-esteem, making the RSES a useful tool in this context. Our first approach was to conduct correlational analyses across our variables to explore the relations evident. Next, given the fact that we have distinct metrics—that is, some trait disposition measures along with measures more tightly coupled to symptomatology—we felt it was important that we examine the influence of each metric on creativity using separate regression analyses.

## 3. Results

Descriptive statistics are presented in Table 1 with correlations among all study variables presented in Table 2.

Our difference score, representing change in the degree to which creative activities were engaged in pre- vs. post-pandemic, showed small positive correlations with metrics of better well-being (i.e., endorsement of positive affective states in the PANAS, increased optimism (LOTr), and self-esteem; Table 2) and small negative correlations with metrics of poor well-being (i.e., endorsing negative affective states on the PANAS, increased boredom proneness, higher scores on the DASS; Table 2). Interestingly, those who reported engaging in more creative pursuits in this early stage of the pandemic were also less likely to break the rules of social distancing. There were some moderate to strong positive relations between rule-breaking and negative affect and symptoms of depression, anxiety, and stress, as well as moderate positive correlations between boredom proneness, negative affect, depression, anxiety, and stress, and a moderate negative correlation with boredom proneness and self-esteem. Finally, self-esteem was strongly negatively correlated with depression, anxiety, and stress, and positively correlated with optimism (i.e., via the Life Orientation Test; Table 2).

Next, we conducted separate regression analyses to determine the extent to which change in the frequency of engaging in creative behaviours predicted well-being on each of our variables of interest. In each model, we also include age, gender, and “pre-COVID activities”—our metric of the sheer number of activities available in a participant’s region—as predictors. Age and gender were included as our past work on affective experiences during the pandemic has shown these to have small, but significant, impacts on our models [5,41]. Note that gender was dummy-coded as male = −1 and female = +1, with participants identifying as other genders omitted from these analyses (this led to the omission of eight participants). As mentioned above, we included a measure of the range of available activities, as any changes post-COVID may be influenced by this factor.

**Figure 1:**
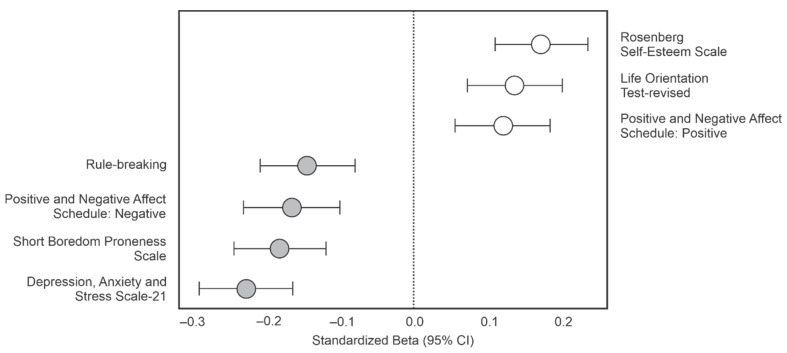
Standardized beta coefficients from the regression models reported in Table 3.

Results demonstrated that changes in the extent to which people engaged in creative endeavours acted as a significant *negative* predictor of the tendency to rule-break and one’s propensity for experiencing boredom. Similarly, the degree to which individuals endorsed negative affective states was negatively predicted by our creative engagement metric; that is, the more frequently people engaged in creative endeavours, the lower their reports of negative affect. In contrast, creative engagement acted as a significant *positive* predictor of positive affective states, higher levels of optimism, and self-esteem (Table 3; Figure 1).

Age consistently functioned in much the same way as creative engagement. That is, age was a positive predictor of well-being (higher positive affect, optimism, and self-esteem) and a negative predictor of factors associated with poorer mental health (e.g., boredom-proneness, negative affect, depression, and anxiety), although it should be noted that the beta weights accounted for a substantially smaller amount of variance relative to the creative engagement variable. Gender was less consistently predictive of well-being. With respect to rule-breaking, men were more likely to rule-break than women and reported higher levels of boredom proneness, consistent with previous work [49]. The only other factor for which gender was a significant predictor was the extent to which positive affective states were reported, with women reporting lower levels of positive affect than men.

## 4. Discussion

An individual’s tendency to engage in more (relative to pre-pandemic levels) everyday creative outlets is a strong predictor of better outcomes for well-being (Table 3 and Figure 1). Although age also functioned in much the same way (with older adults demonstrating better well-being), creative outlets accounted for unique variance in all our regression models. The manner in which creative pursuits normally promote well-being is likely multifactorial [27,50]. In the context of the pandemic—a circumstance in which extraordinary measures curtailed our sense of autonomy—it may be the case that engaging in creative pursuits promotes well-being by helping to re-establish feelings of agency and control. On the one hand, a lack of autonomy has been shown to stifle creativity in the classroom and at work (e.g., [51,52,53]), while a sense of autonomy itself has been shown to promote well-being [54]. While the current dataset cannot definitively isolate the mechanistic factor at play here, promoting one’s sense of agency/autonomy seems a plausible candidate. In addition, agency has been suggested as a key factor in boredom-proneness, such that those high in boredom-proneness feel a loss of agency [55]. Certainly, in the current dataset, boredom-proneness was consistently associated with poor well-being and lower levels of engagement in creative outlets (Table 2).

As mentioned in the Introduction, prior research on creativity has suggested that successful pursuit of creative outlets depends less on social aspects of an activity and more on an individual’s playfulness and capacity for inspiration [26]. While neither the work of Secker and colleagues [26] nor the current study would suggest there is no influence of social factors on creativity, the current findings do suggest that we can extract positive outcomes from being creative while in isolation. Furthermore, doing so may have made it easier for people to adhere to the rules of social distancing (Table 3A and Figure 1).

An important caveat to these findings concerns the amount of free time an individual has. Those who were primary caregivers during the pandemic likely had less time available to pursue creative outlets. Our current dataset does not allow for a direct test of the notion that available time was critical in promoting creativity. Some other aspects regarding self-regulation suggest that whatever role time did play, it was not a singular driving factor. That is, those who were more prone to boredom were less likely to engage in creative outlets (Table 3B and Figure 1). It is difficult to imagine that the boredom-prone exhibit this relation as a function of how much time they do or do not have available. Instead, they are more likely to be simply failing to launch into activities, creative or otherwise, that would help promote their sense of agency and well-being [47].

Beyond establishing a sense of agency, there are many potential reasons behind the therapeutic benefits of creative outlets. As mentioned in the Introduction, the notion that such outlets foster a sense of playfulness and inspiration may in turn promote positive affect [26]. In addition, the often intrinsically motivated nature of creative activities [27,56], and the connection between creativity and the state of flow, may each promote positive affect [57]. Finally, pursuing creative activities can be construed as a meaning-making exercise [58]. Certainly, the negative relation here with boredom-proneness supports this notion. That is, those who are prone to boredom have a high need for meaning in their lives [46]—a need that frequently is unfulfilled, driving their boredom in a kind of vicious cycle. Here, those who were capable of engaging creatively with their environs tended to be lower in boredom-proneness (Table 2). As such, the meaning derived from their creative pursuits may be a critical factor in their positive well-being.

Cultural factors may have played a role in the current dataset. In one study of Italian young adults, the extent to which participants endorsed a collectivist viewpoint was related to better adjustment to the restrictions imposed by the pandemic [59]. One recent study showed that those who hold more collectivistic views demonstrated a stronger relation between the positive coping effects of creativity, flourishing, and social well-being during the pandemic [60]. Given that the public health messaging during the pandemic was commonly focused on personal responsibility (at least in North America, where this sample was collected), it may be important for public policymakers to consider a different approach. Rather than focusing on the *constraints* individuals are being asked to abide by, it may prove more useful to focus instead on the *potential activities* that we know will promote well-being in such extraordinary times.

## 5. Limitations

We have measured creativity here in a quantitative manner. This, of course, neglects the qualitative aspects of creative pursuits, which may also provide insights into an individual’s state of mind. This could be achieved in a manner similar to studies that examine the content and quality of thought processes evoked during episodes of mind-wandering [61]. Exploring the qualitative aspects of creative outputs in this way may lead to important insights into the consequences of the restrictions imposed by the pandemic on well-being. In addition, this is a snapshot of a single point in time, captured early in the pandemic. Whether the pursuit of creative activities leads to *sustained* benefits to well-being, as well as whether there are bidirectional effects of well-being on creativity, are important open questions. Furthermore, we have not considered other aspects of individuals’ lives that may have impacted their capacity to engage in creative pursuits. For instance, working from home may provide more opportunities to engage in various activities that would not be feasible for those who were required to go into their workplace. It is also worth pointing out that the aspects of well-being examined here are clearly not exhaustive. Indeed, our definition of well-being encompasses both challenges with mental health (e.g., depressive and anxiety symptoms assessed via the DASS), as well as factors one might characterize as a “sense of purpose, meaning, and optimism” captured in trait levels of optimism and self-esteem. Clearly, this is both a mixture of constructs, and a non-exhaustive one at that. As just one example of other domains worth considering, there is work showing the importance of social connectivity and loneliness [62], suggestive of at least one important facet of pandemic experiences that could have impacted our results. Finally, our results could have connected more directly with findings from the positive psychology literature [58,63,64,65]. Others have already made the call for research exploring how aspects of positive psychology—ranging from meaning-making, self-compassion, and gratitude, to factors affecting the quality of our interpersonal relations—might help buffer against the effects of the pandemic [64,65]. In a recent survey study, results showed that people endorsed a wide variety of positive psychology concepts including empathy, service, and gratitude, as ways to help cope with the strictures of the pandemic [65]. It is plausible that engaging in creative practices of the kind assessed here (e.g., making gift cards for friends and loved ones) directly connects to many of those concepts. Certainly, future research could examine this link between creativity and positive psychology to further develop optimal prescriptions for coping in the pandemic. What the current results make clear is that there are important benefits to be gained from engaging in creative pursuits when the ease of access to goal pursuits, more generally, has been constrained.

## Figures and Tables

**Table 1:** Descriptive statistics.

Measure	Mean	SD	Skew	Kurtosis	Cronbach’s Alpha
Creative Activities	0.19	0.26	0.21	3.18	-
Availability of All Activities Pre-COVID	62.31	26.54	−0.57	2.37	0.96
Rule Breaking	0.01	1.00	2.12	7.24	-
Short Boredom Proneness Scale	3.52	1.52	0.11	2.06	0.92
Positive and Negative Affect Schedule: Positive	29.46	8.96	0.16	2.36	0.91
Positive and Negative Affect Schedule: Negative	19.32	9.42	1.10	3.26	0.94
Depression, Anxiety and Stress Scale-21	35.39	15.02	0.97	2.83	0.97
Life Orientation Test-revised	20.63	6.62	−0.40	2.31	0.92
Rosenberg Self-Esteem Scale	6.52	1.88	−0.57	2.53	0.94

**Table 2:** Correlation matrix for all study variables.

	2.	3.	4.	5.	6.	7.	8.	9.
1. Creative Activities	0.04	−0.13 *	−0.17 *	0.12 *	−0.16 *	−0.22 *	0.13 *	0.17 *
2. Availability of All Activities Pre-COVID		−0.02	−0.08 *	0.14 *	−0.04	−0.08 *	0.12 *	0.14 *
3. Rule Breaking			0.36 *	0.28 *	0.55 *	0.6 *	−0.14 *	−0.29 *
4. Short Boredom Proneness Scale				−0.24 *	0.58 *	0.63 *	−0.43 *	−0.52 *
5. Positive and Negative Affect Schedule: Positive					−0.01	−0.03	0.39 *	0.35 *
6. Positive and Negative Affect Schedule: Negative						0.85 *	−0.38 *	−0.53 *
7. Depression, Anxiety and Stress Scale−21							−0.45 *	−0.64 *
8. Life Orientation Test-revised								0.70 *
9. Rosenberg Self-Esteem Scale								

* *p* < 0.05.

**Table 3:** Regression models predicting well-being.

A. Rule-Breaking					
	Predictors	Beta	95% CI	*t*-value	*p*-value
	(Intercept)	0.48	0.19–0.76	3.28	0.001 **
	Age	−0.01	−0.02–0.00	−3.10	0.002 **
	Gender	−0.16	−0.22–−0.09	−4.62	<0.001 ***
	Availability of All Activities Pre-COVID	0.00	0.00–0.00	−0.47	0.638
	Creative Activities	−0.55	−0.79–−0.30	−4.38	<0.001 ***
	R^2^/R^2^ adjusted	0.059/0.054			
B. Short Boredom Proneness Scale					
	Predictors	Beta	95% CI	*t*-value	*p*-value
	(Intercept)	5.25	4.83–5.68	24.28	<0.001 ***
	Age	−0.03	−0.04–−0.02	−7.38	<0.001 ***
	Gender	−0.12	−0.22–−0.03	−2.50	0.013 *
	Availability of All Activities Pre-COVID	0.00	−0.01–0.00	−2.59	0.01 **
	Creative Activities	−1.05	−1.41–−0.69	−5.68	<0.001 ***
	R^2^/R^2^ adjusted	0.107/0.103			
C. Positive and Negative Affect Schedule: Positive					
	Predictors	Beta	95% CI	*t*-value	*p*-value
	(Intercept)	22.36	19.78–24.94	17.01	<0.001 ***
	Age	0.09	0.03–0.14	3.24	0.001 **
	Gender	−0.67	−1.26–−0.07	−2.20	0.028 *
	Availability of All Activities Pre-COVID	0.05	0.02–0.07	4.16	<0.001 ***
	Creative Activities	4.08	1.87–6.28	3.62	<0.001 ***
	R^2^/R^2^ adjusted	0.047/0.043			
D. Positive and Negative Affect Schedule: Negative					
	Predictors	Beta	95% CI	*t*-value	*p*-value
	(Intercept)	26.38	23.68–29.08	19.17	<0.001 ***
	Age	−0.13	−0.19–−0.08	−4.81	<0.001 ***
	Gender	−0.44	−1.06–0.19	−1.37	0.17
	Availability of All Activities Pre-COVID	−0.01	−0.04–0.01	−1.10	0.272
	Creative Activities	−5.94	−8.26–−3.63	−5.05	<0.001 ***
	R^2^/R^2^ adjusted	0.057/0.053			
E. Depression, Anxiety and Stress Scale−21					
	Predictors	Beta	95% CI	*t*-value	*p*-value
	(Intercept)	50.81	46.61–55.02	23.72	<0.001 ***
	Age	−0.27	−0.35–−0.18	−6.13	<0.001 ***
	Gender	−0.87	−1.84–−0.1	−1.75	0.08
	Availability of All Activities Pre-COVID	−0.04	−0.08–−0.01	−2.46	0.014 *
	Creative Activities	−12.97	−16.57–−9.37	−7.08	<0.001 ***
	R^2^/R^2^ adjusted	0.101/0.097			
F. Life Orientation Test-revised					
	Predictors	Beta	95% CI	*t*-value	*p*-value
	(Intercept)	14.00	12.12–15.89	14.56	<0.001 ***
	Age	0.11	0.07–0.14	5.41	<0.001 ***
	Gender	0.20	−0.23–0.64	0.92	0.357
	Availability of All Activities Pre-COVID	0.03	0.01–0.05	3.79	<0.001 ***
	Creative Activities	3.44	1.83–5.06	4.18	<0.001 ***
	R^2^/R^2^ adjusted	0.067/0.063			
G. Rosenberg Self-Esteem Scale					
	Predictors	Beta	95% CI	*t*-value	*p*-value
	(Intercept)	4.43	3.90–4.96	16.38	<0.001 ***
	Age	0.03	0.02–0.04	5.95	<0.001 ***
	Gender	0.08	0.04–0.21	1.36	0.175
	Availability of All Activities Pre-COVID	0.01	0.01–0.01	4.24	<0.001 ***
	Creative Activities	1.23	0.78–1.69	5.32	<0.001 ***
	R^2^/R^2^ adjusted	0.089/0.084			

* *p* < 0.05, ** *p* < 0.01, *** *p* < 0.001.

## Data Availability

All data used in this study are available from the following Open Science Framework link: https://osf.io/dmk9s/, accessed on 9 June 2021).
